# Validation of claims data to identify death among aged persons utilizing enrollment data from health insurance unions

**DOI:** 10.1186/s12199-019-0819-3

**Published:** 2019-11-23

**Authors:** M. Sakai, S. Ohtera, T. Iwao, Y. Neff, G. Kato, Y. Takahashi, T. Nakayama, on behalf of BiDAME (Big Data Analysis of Medical care for the Elderly in Kyoto)

**Affiliations:** 1 Department of Health Informatics, Kyoto University School of Public Health, Yoshidakonoe-cho, Sakyo-ku Kyoto, 606-8501 Japan; 2 Division of Medical Information Technology and Administration Planning, Kyoto University Hospital, 54 Kawahara-cho, Shogoin, Sakyo-ku Kyoto, 606-8507 Japan; 3 Department of Research, Institute for Health Economics and Policy, Tokyo, Japan; 4 Solutions Center for Health Insurance Claims, Kyoto University Hospital, 54 Kawahara-cho, Shogoin, Sakyo-ku Kyoto, 606-8507 Japan

**Keywords:** Aged, Health insurance claims, Sensitivity, Specificity, Validation

## Abstract

The identification of death is critical for epidemiological research. Despite recent developments in health insurance claims databases, the quality of death information in claims is not guaranteed because health insurance claims are collected primarily for reimbursement. We aimed to examine the usefulness and limitations of death information in claims data and to examine methods for improving the quality of death information for aged persons.

We used health insurance claims data and enrollment data (as the gold standard) from September 2012 through August 2015 for nondependent persons aged 65–74 years enrolled in Japanese workplace health insurance. Overall, 3,710,538 insured persons were registered in the database during the study period. We analyzed 45,441 eligible persons. Inpatient and outpatient deaths were identified from the discharge/disease status in the claims, with sensitivities of 94.3% and 47.4%, specificities of 98.5% and 99.9%, and PPVs of 96.3% and 95.7%, respectively, using enrollment data as the gold standard. For outpatients, death defined as a combination of disease status and charge data for terminal care still indicated low sensitivity (54.7%).

The validity of death information in inpatient claims was high, suggesting its potential usefulness for identifying death. However, given the low sensitivity for outpatient deaths, the use of death information obtained solely from records in outpatient claims is not recommended.

## Introduction

In Japan, an ultra-aging, high-mortality society, 33.0% of the population, were ≥ 60 years old in 2017 [1]. Deceased persons in Japan comprised approximately 1,200,000 in 2014; this is estimated to increase to approximately 1,670,000 by 2040 [2]. In constructing a sustainable end-of-life care system, several challenges for research have emerged, surveying mortality and medical care practice for aged persons at the end of life [3–5]. Thus, the identification of death is critical for epidemiological research targeting aged persons.

Recent developments in the health insurance claims databases of government agencies and the private sector have transformed epidemiological research in Asia-Pacific countries [6–12]. In Japan, private companies have created health insurance claims databases for research. The National Database of Health Insurance Claims and Specific Health Checkups of Japan (NDB) that contains almost 100% of the digitized health insurance claims for the entire country was also constructed.

However, health insurance claims data are collected primarily for reimbursement, rather than for research following the patient prognosis. Thus, quality of death information in claims is not guaranteed. Previous study has examined the validity of death information recorded in claims using data in 2005–2009 and indicated that the sensitivity of death information in claims was low [13]. However, to date, a method to improve the validity of death information in claims has not been established. Currently, validation studies using the latest claims database are underway [14]. As part of this effort, we aimed to examine usefulness and limitations when using death information in claims databases and to examine methods for improving the quality of death information for aged persons.

## Methods

### Study design

This cross-sectional study validated death information from health insurance claims against that recorded in enrollment data for the health insurance union from the same month (the latter serving as a gold standard). Because the claims data used in the present study did not include information on the date of death, the data were compared by month.

### Data source

Claim validation requires the linkage of claims data and other sources of highly reliable data (gold standard). However, The Japanese Ministry of Health, Labor and Welfare (MHLW) prohibits linking data from NDB to external data. Hence, we utilized workplace health insurance’s claims database which directly link health insurance claims and enrollment data at the individual patient level with high precision [15]. We used the claims database for insured persons enrolled in workplace health insurance unions that were available through a database vendor, Japan Medical Data Center Co., Ltd. This database contains monthly claims submitted to health insurance unions, particularly for those insured in Japanese health insurance unions for employees of large companies (union-managed health insurance). As of September 2015, these comprised approximately 10% of all Japanese beneficiaries. The database does not include the data of those enrolled in health insurance unions targeting medium-sized to small businesses, seamen, public employees, self-employed individuals, and those covered by the Medical Insurance System for individuals aged ≥ 75 years.

The data provided the discharge status for inpatient claims, with the following potential values: “continued,” “cure,” “death,” “termination,” and “transferred.” Values reflect the status of healthcare provision: “continued” represents continued therapy; “cure” indicates that no further healthcare was needed due to complete cure or improvement; “termination” suggests that no healthcare will be provided at least for the time being; and “transferred” indicates that the patient was transferred to another hospital. The same information was also available as the disease status in the outpatient claims database.

The enrollment data recorded the month and year of the loss of insurance status as well as the reason for the loss of insured status (“retired, moved away, died, term expired, insurance premiums unpaid, transition, household separated, or other”). When an insured individual loses their insured status, the employer notifies the union, and this is reflected in the enrollment data.

### Study participants

We included nondependent insured persons aged 65–74 years registered in the workplace health insurance’s claims database between October 2012 and September 2015. We analyzed only nondependent insured persons to guarantee gold standard-level accuracy in death information from enrollment data. The insurance status for dependents is sometimes misclassified (in some cases, if the insured individual dies, the dependent of that insured individual is also registered as dead) and thus dependents are excluded from analysis. To conduct validation in a cross-sectional study design that would compare claims from the same month with enrollment data from the health insurance union, we excluded patients with missing information on health insurance union enrollment status (i.e., continued enrollment/loss of insured status) for the month of the most recent claim data (i.e., claims issued most recently). We also excluded any of those who lost their insured status with unknown reasons for the loss of insured status.

### Validation of death information that can be obtained from claims

#### Claims-based definition of death

We defined claims-based definition of death as patients for whom the discharge/disease status recorded in the most recent (last issued) claims was death (Definition 1). If multiple claims were issued in the same month, all claims were examined. If at least one claim noted death as the discharge/disease status, the patient was considered deceased. As we do not intend to develop a system by which health insurance associations confirm death from claims, we examined methods for increasing the validity of death information for outpatients by combining disease status and charge data recorded in claims. If two or more house calls or home visits are made within 14 days of death, a fee for terminal care can be charged for outpatients. Fees can also be charged when death certificates are issued at a patient’s residence. We included these 2 charges for the definition of outpatient death to improve the validity. Definition 2 applied to patients for whom no claims were issued after a fee was charged for terminal care. Definition 3 applied to patients for whom no claims were issued after a death certificate was charged. Definition 4 applied to patients for whom the outpatient disease status was recorded as death or no claims were issued after a fee was charged for terminal care, or a death certificate was issued (i.e., Definition 1 or 2 or 3).

### Gold standard definition of death from the enrollment data

We defined the gold standard deceased patients as those for whom the loss of the insured status due to death was recorded in the health insurance union enrollment data. Patients, whose enrollment data listed the reason for the loss of the insured status as a cause other than death or were in a “continued” status, were not regarded as gold standard deceased patients.

### Defining true positives, false negatives, false positives, and true negatives

True positives were defined as cases with any claims-based definition of death (i.e., death information can be obtained from claims) and gold standard definition of death (i.e., the reason for the loss of the insured status in the enrollment data was recorded as death). False negatives were defined as cases with no claims-based definition of death but with a gold standard definition of death. False positives were defined as cases with any claims-based definition of death but not the case with the gold standard definition of death (i.e., the reason for the loss of the insured status in the enrollment data listed a cause other than death or was in the “continued” status). True negatives were defined as cases with no claims-based definition of death and no gold standard definition of death.

### Statistical analysis

#### Sensitivity, specificity, and positive predictive value

We calculated the sensitivity, specificity, and positive predictive value (PPV) of our claims-based definitions of death for inpatients and outpatients separately. In the present validation, only claims issued in the most recent month for each patient were analyzed; thus, inpatients and outpatients were categorized based on claims issued in the most recent month. Inpatients were those who had received inpatient medical care during the most recent month of the issued claim (i.e., patients with 1+ inpatient claims issued), and inpatient claims data were analyzed. Outpatients were those who only received outpatient medical care during the most recent month of the issued claim (i.e., those for whom only outpatient claims were issued), and their outpatient claims data were analyzed.

R version 3.2.4 was used for statistical analysis. Informed consent was not obtained because our study only used data that were anonymized in an unlinkable fashion (the data were anonymized using a method that does not leave a lookup table linking a patient with an assigned code or number to prevent the identification of specific patients). The study protocol was approved by Kyoto University’s research ethics committee.

## Results

### Patient characteristics

Overall, 3,710,538 insured persons were registered in the database from October 2012 through September 2015. We analyzed 45,441 nondependent insured persons (43,870 outpatients and 1571 inpatients), excluding 3,584,302 persons aged < 65 years, 56,130 dependents, 5743 persons with no health insurance claims, 13,370 persons with no data on health insurance union enrollment status (i.e., continued enrollment/loss of insured status), and 5552 persons with unknown reasons for losing their insured status.

Sex, age, year of enrollment in the health insurance union, enrollment period, and presence/absence of insured status in the union are shown in Table 1 for all cases subjected to analysis. We analyzed nondependent insured persons to guarantee gold standard-level accuracy in death information from enrollment data. Thus, both inpatients and outpatients comprised a relatively high proportion of males. The median enrollment duration in a health insurance union was 115 and 101 months for inpatients and outpatients, respectively. Inpatients and outpatients who lost their insured status between October 2012 and September 2015 numbered 796 (50.7%) and 13,042 (29.7%), respectively. Of these, inpatients and outpatients who lost their insured status due to death numbered 473 (30.1%) and 95 (0.2%), respectively.

**Table 1:** Patient characteristics

	Total (*N* = 45,441)		Inpatients (*N* = 1571)		Outpatients (*N* = 43,870)	
	*N*	%	*N*	%	*N*	%
Sex						
Male	40,846	89.9	1500	95.5	39,346	89.7
Female	4595	10.1	71	4.5	4524	10.3
Age, years						
65–69	31,853	70.1	924	58.8	30,929	70.5
70–74	13,588	29.9	647	41.2	12,941	29.5
Number of claims issued in the most recent month						
1	32,084	70.6	1404	89.4	30,680	69.9
2 or more	13,357	29.4	167	10.6	13,190	30.1
Year of enrollment in a health insurance union						
2000 or earlier	9282	20.4	337	21.5	8945	20.4
2001–2005	11,245	24.7	500	31.8	10,745	24.5
2006–2010	14,984	33.0	485	30.9	14,499	33.0
2011–2015	9930	21.9	249	15.8	9681	22.1
Enrollment period, months (median)	89		115		101	
Union enrollment status						
Continued enrollment	31,603	69.5	775	49.3	30,828	70.3
Loss of insured status	13,838	30.5	796	50.7	13,042	29.7
Reason for loss of insured status						
Retirement	6723	14.8	176	11.2	6547	14.9
Relocation	104	0.2	2	0.1	102	0.2
Unpaid insurance fees	1130	2.5	12	0.8	1118	2.5
Other	5313	11.7	133	8.5	5180	11.8
Death	568	1.2	473	30.1	95	0.2

### Validation of claims-based definition of death

Table 2 shows results of the validation of death based on claims, with health insurance enrollment data regarded as the gold standard. Regarding the definition using the information of discharge or disease status only (Definition 1, Table 2), the sensitivity, specificity, and PPV were 94.3% (446/473), 98.5% (1081/1098), and 96.3% (446/463) for inpatients, and 47.4% (45/95), 99.9% (43,773/43,775), and 95.7% (45/47) for outpatients, respectively. Among outpatients, regarding the definition using reimbursements for terminal care (Definition 2), the sensitivity, specificity, and PPV were 37.9% (36/95), 100.0% (43,775/43,775), and 100.0% (36/36), respectively. Regarding the definition using the issuance of death certificates at home (Definition 3), the sensitivity, specificity, and PPV were 9.5% (9/95), 100.0% (43,775/43,775), and 100.0% (9/9), respectively. When the reimbursement claims for the issuance of a death certificate and terminal care were combined with the disease status (Definition 4), the cases of death were identified with a sensitivity, specificity, and PPV of 54.7% (52/95), 100.0% (43,775/43,775), and 100.0% (52/52), respectively.

**Table 2:** Sensitivity and specificity of the claims-based definition of death

Definition	Description	Total	True positive	False positive	False negative	True negative	Sensitivity		Specificity		Positive predictive value (PPV)	
Inpatients												
1	Death as discharge status	1571	446	17	27	1081	94.3	[92.2–96.4]	98.5	[97.7–99.2]	96.3	[94.6–98.0]
Outpatients												
1	Death as disease status	43,870	45	2	50	43,773	47.4	[37.3–57.4]	99.9	[99.9–100.0]	95.7	[90.0–100.0]
2	Terminal care at home + claims terminated	43,870	36	0	59	43,775	37.9	[28.1–47.7]	100.0	[100.0–100.0]	100.0	[100.0–100.0]
3	Death certificate + claims terminated	43,870	9	0	86	43,775	9.5	[3.6–15.4]	100.0	[100.0–100.0]	100.0	[100.0–100.0]
4	1 or 2 or 3	43,870	52	0	43	43,775	54.7	[44.7–64.7]	100.0	[100.0–100.0]	100.0	[100.0–100.0]

## Discussion

We examined usefulness and limitations of death information in claims and methods for improving the quality of death information for aged persons. For inpatients, both the sensitivity (94.3%) and specificity (98.5%) of death information in the claims were high. Among outpatients, however, the specificity was high (99.9%), but the sensitivity was low (47.4%) (Table 2). The addition of reimbursement claims for terminal care or the issuance of a death certificate at home to the definition of death, followed by the termination of subsequent health insurance claims, still indicated low sensitivity (54.7%) (Table 2).

Despite developments in health insurance claims databases, the quality of death information in claims is not guaranteed because those data are collected primarily for reimbursement. A previous validation study using the claims data in Japan between January 2005 and August 2009 reported that the sensitivity of death was limited [13]. The increase in validity among inpatients compared with the previous study may be due to policies promoting digitization, such as the obligation to submit health insurance claims online starting in 2011 [16]. Differences in inpatient characteristics between the 2 study populations (20–74 years in Ooba et al.’s study [13] and 65–74 years in our study) also likely contributed to the discrepancy in the results. On the other hand, validity among outpatients still remained low compared with the previous study [13]. Possible reasons for the low sensitivity of outpatient death are attributable to the nature of claims. Because health insurance claims data are collected primarily for reimbursement, medical institutions are not motivated to record deaths in claims, and thus, the omission of recording of death is possible. Additionally, there is no system to follow patients’ prognoses and reflect them in the claims database.

Our study contributed to promote epidemiological research using claims database by increasing understanding for the limitation and usefulness of data. Specificity of death information for both inpatients and outpatients was high, and thus, the overestimation of the number of death due to misclassification of outcomes, that is, researchers misclassify survivors as decedents, is low. High sensitivity of inpatient deaths suggests the potential usefulness for identifying death. The risk of the misclassification of outcomes, that is, researchers misclassify deceased persons as being alive due to the absence of a record of death in the claims, is low. However, it should be noted that 5.7% of deceased persons are possibly misclassified. Given the low sensitivity for outpatient deaths, there is a distinct limitation for identifying death from claims data. Although we also examined methods for increasing the sensitivity of death information by combining disease status and charge data recorded in claims (when death was defined solely by disease status or together with charge records of terminal care), the sensitivity for identifying deaths is still limited. Currently, we recommend not using death information obtained solely from records in outpatient claims. These findings should be known among researchers and health insurance societies when they use claims data.

This study possessed some limitations. Regarding the generalization of our results, the target population comprised nondependent insured persons aged 65–74 years enrolled in workplace health insurance; thus, our results may not be applicable to all aged persons. Moreover, subjects were enrolled in health insurance unions insuring a fraction of all large companies. We did not include subjects enrolled in health insurance unions targeting medium-sized to small businesses, seamen, public employees, self-employed individuals, and individuals covered by the Medical Insurance System for individuals ≥ 75 years. Finally, although the PPV depends on the prevalence (i.e., prior probability of death) of the study population, we know little about the prevalence of our study population. Therefore, our findings cannot be applied to the claims data of all aged persons. Nevertheless, the claims database used in the present study represents the best available current data because validation to identify death in aged persons could be performed by the direct linkage of health insurance claims and health insurance enrollment data.

## Conclusions

We examined usefulness and limitations of using death information in claims databases and examined methods to improve the quality of death information for aged persons. High sensitivity and specificity of death information in inpatient claims suggested the potential utility of identifying death. However, given the low sensitivity for outpatient deaths, the use of death information obtained solely from records in outpatient claims is not recommended.

## Data Availability

Not applicable

## Abbreviations

BiDAME: Big Data Analysis of Medical care for the Elderly in Kyoto
NDB: National Database of Health Insurance Claims and Specific Health Checkups of Japan
